# Natural Product Chemistry of Gorgonian Corals of Genus *Junceella*–Part III

**DOI:** 10.3390/md16090339

**Published:** 2018-09-17

**Authors:** Hsu-Ming Chung, Yi-Chen Wang, Chung-Chih Tseng, Nan-Fu Chen, Zhi-Hong Wen, Lee-Shing Fang, Tsong-Long Hwang, Yang-Chang Wu, Ping-Jyun Sung

**Affiliations:** 1 Department of Applied Chemistry, National Pingtung University, Pingtung 900, Taiwan; shiuanmin@mail.nptu.edu.tw; 2 Division of Cardiology, Department of Internal Medicine, Kaohsiung Armed Forces General Hospital, Kaohsiung 802, Taiwan; cvyc.wang@gmail.com; 3 Department of Marine Biotechnology and Resources, National Sun Yat-sen University, Kaohsiung 804, Taiwan; caviton@gmail.com (C.-C.T.); wzh@mail.nsysu.edu.tw (Z.-H.W.); 4 Division of Dentistry, Zuoying Branch of Kaohsiung Armed Forces General Hospital, Kaohsiung 813, Taiwan; 5 Division of Neurosurgery, Department of Surgery, Kaohsiung Armed Forces General Hospital, Kaohsiung 802, Taiwan; chen06688@gmail.com; 6 Department of Neurological Surgery, Tri-Service General Hospital, National Defense Medical Center, Taipei 114, Taiwan; 7 Center for Environmental Toxin and Emerging-Contaminant Research, Cheng Shiu University, Kaohsiung 833, Taiwan; lsfang@csu.edu.tw; 8 Super Micro Mass Research and Technology Center, Cheng Shiu University, Kaohsiung 833, Taiwan; 9 Graduate Institute of Natural Products, College of Medicine, Chang Gung University, Taoyuan 333, Taiwan; 10 Chinese Herbal Medicine Research Team, Healthy Aging Research Center, Chang Gung University, Taoyuan 333, Taiwan; 11 Research Center for Chinese Herbal Medicine, Research Center for Food and Cosmetic Safety, Graduate Institute of Healthy Industry Technology, College of Human Ecology, Chang Gung University of Science and Technology, Taoyuan 333, Taiwan; 12 Department of Anaesthesiology, Chang Gung Memorial Hospital, Taoyuan 333, Taiwan; 13 Graduate Institute of Natural Products, Kaohsiung Medical University, Kaohsiung 807, Taiwan; 14 Research Center for Natural Products and Drug Development, Kaohsiung Medical University, Kaohsiung 807, Taiwan; 15 Department of Medical Research, Kaohsiung Medical University Hospital, Kaohsiung 807, Taiwan; 16 Chinese Medicine Research and Development Center, China Medical University Hospital, Taichung 404, Taiwan; 17 National Museum of Marine Biology and Aquarium, Pingtung 944, Taiwan; 18 Graduate Institute of Marine Biology, National Dong Hwa University, Pingtung 944, Taiwan

**Keywords:** *Junceella*, gorgonian, briarane, biomedical activity

## Abstract

The structures, names, bioactivities, and references of 82 natural products, including 48 new metabolites, purified from the gorgonian corals belonging to the genus *Junceella* are described in this review. All compounds mentioned in this review were obtained from *Junceella fragilis*, *Junceella gemmacea*, *Junceella juncea*, and *Junceella* sp., collected from tropical Indo-Pacific Ocean. Some of these compounds exhibited potential biomedical activities.

## 1. Introduction

Following previous review articles focused on marine-origin natural products, this review covers the literature from October 2011 to August 2018, and describes 82 natural products (including 48 new metabolites) from gorgonian corals belonging to the genus *Junceella* (family Ellisellidae) [1,2,3,4]. Extending from previous reviews in 2004 and 2011 [5,6], this review provides structures, names, bioactivities, and references for all compounds including briarane- and norcembrane-type diterpenoids, sterol, and nucleosides. Many of these compounds exhibited interesting bioactivities in vitro, which might indicate a potential for use in biomedical applications. This survey of natural products from *Junceella* is presented taxonomically according to species. 

## 2. Junceella

### 2.1. Junceella Fragilis

**Figure 1:**
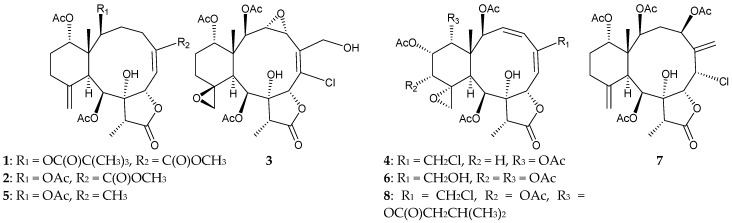
Structures of frajunolides P–S (**1**–**4**), umbraculolide A (**5**), juncenolide C (**6**), junceellonoid A (**7**), and juncin R (**8**).

Eight 8-hydroxybriaranes, including four new compounds, frajunolides P–S (**1**–**4**), and four known metabolites, umbraculolide A (**5**) [7,8], juncenolide C (**6**) [9], junceellonoid A (**7**) [10], and juncin R (**8**) [11], were isolated from *J. fragilis*, collected from the waters of Taiwan [12] (Figure 1). The structures of briaranes **1**–**4** were established by spectroscopic methods, and determination of the absolute configuration of **1** was completed by X-ray diffraction analysis [12]. At a concentration of 10 μg/mL, briaranes **1** and **2** were found to exert inhibitory activities on elastase release (inhibition rate = 35.6% and 34.1%, respectively) and superoxide anion production (inhibition rate = 32.5 and 28.7%, respectively) by human neutrophils [12].

**Figure 2:**
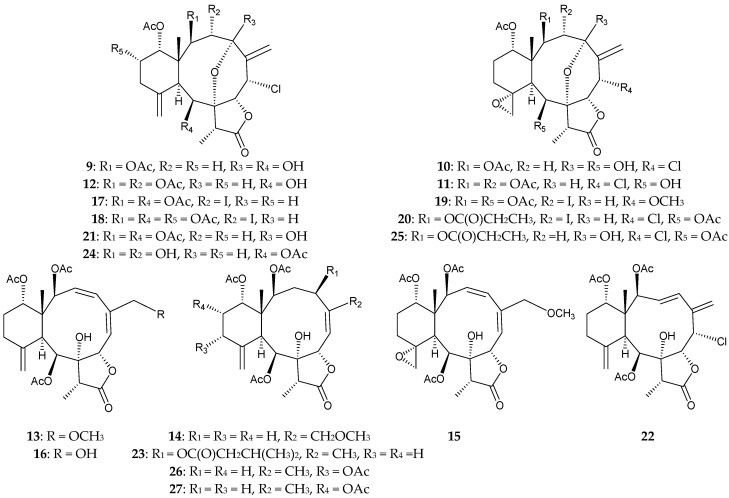
Structures of fragilisinins A–L (**9**–**20**), junceellolides A (**21**) and B (**22**), junceol A (**23**), junceellonoid D (**24**), fragilide C (**25**), and frajunolides A (**26**) and E (**27**).

In 2014, *J. fragilis*, collected from the South China Sea, was found to contain 12 new briaranes, fragilisinins A–L (**9**–**20**) [13], along with seven known analogues, junceellolides A (**21**) and B (**22**) [14], junceol A (**23**) [15], junceellonoid D (**24**) [16,17], fragilide C (**25**) [18], and frajunolides A (**26**) [19] and E (**27**) [20] (Figure 2) [13]. The structures of briaranes **9**–**20** were determined by spectroscopic methods. Briaranes **17**–**20** were the first iodine-containing briaranes to be isolated. The absolute configuration of briarane **9** was confirmed by single-crystal X-ray diffraction data [13]. Briaranes **13**, **14**, **18**, **21**, and **24** showed potent antifouling activities against the settlement of barnacle *Balanus amphitrite* larvae, with EC_50_ values of 14.0, 12.6, 11.9, 5.6, and 10.0 μM, respectively [13].

**Figure 3:**
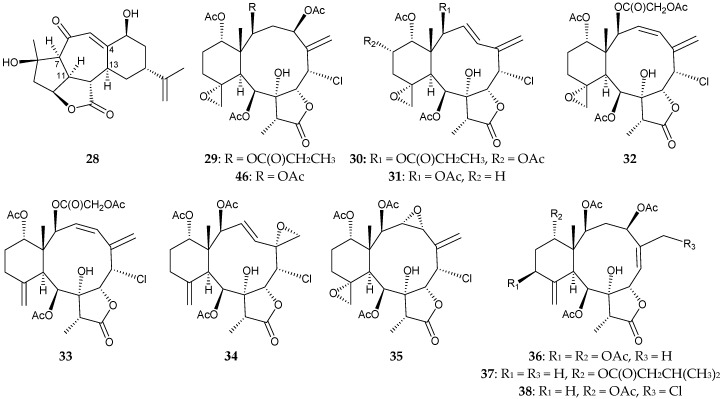

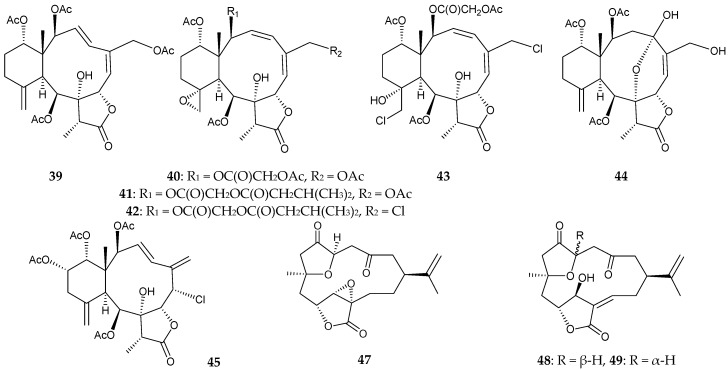
Structures of fragilolides A–Q (**28**–**44**), frajunolides H (**45**) and N (**46**), scabrolide D (**47**), sinuleptolide (**48**), and 5-*epi*-sinuleptolide (**49**).

In addition, a new norditerpenoid, fragilolide A (**28**), 16 new briaranes, fragilolides B–Q (**29**–**44**), along with two known briaranes, frajunolides H (**45**) [20] and N (**46**) [21], and three known norcembranoids, scabrolide D (**47**) [22], sinuleptolide (**48**) [23], and 5-*epi*-sinuleptolide (**49**) [24,25,26], were obtained from *J. fragilis*, collected from the inner coral reef around in Hainan Island of China [27] (Figure 3). The structures of metabolites **28**–**44** were determined by spectroscopic methods, including calculated electronic circular dichroism (ECD) data. The structures, including the absolute configurations of briaranes **37** and **46**, were further established by single-crystal X-ray diffraction analysis using Flack parameter in this study [27]. Compound **28** featured an unprecedented 4,13- and 7,11-fused tetracyclic norcembranoid [27].

Briarane **45** exhibited cytotoxicity toward Hep G2 (human hepatocellular carcinoma), Huh7 (human hepatocellular carcinoma), SMMC-7721 (human papillomavirus-related endocervical adenocarcinoma), A2780 (human ovarian carcinoma), BGC-823 (human gastric adenocarcinoma), HGC-27 (human gastric carcinoma), MGC-803 (human gastric carcinoma), NCI-H1650 (human bronchoalveolar carcinoma), and PA-1 (human ovarian mixed germ cell tumor) cells with IC_50_ values 0.89, 16.52, 0.61, 1.18, 2.10, 0.61, 1.97, 6.47, and 0.42 μM, respectively. Briaranes **31**, **34**, **36**, **39**, **43**, and **46** exerted selective inhibitory effects toward hepatitis B and antigen (HBeAg) in a dose of 10 μM, whereas no activity was observed against the expression of hepatitis B surface antigen (HBsAg) [27].

Moreover, the norcembranoids **28** and **47**–**49** were assayed for their potential inhibitory effects against nitric oxide (NO) production induced by lipopolysaccharides (LPS) (large molecules consisting of lipids and polysaccharide composed of *O*-antigen joined by chemical bonds), in RAW264.7 cells, and these four compounds exhibited the inhibitory activities with 27.8%, 43.5%, 56.0%, and 57.9% inhibition, respectively, at a dose of 100 μM [27]. In order to explore the mechanism of these NO inhibitors, the expression of the antioxidant response element (ARE) mediated lufiferase and NF-κB was evaluated. Norcembranoids **48** and **49** showed the effects against NF-κB by the inhibitory rates of 25.1% and 28.6% in a dose of 50 μM, respectively. Significant induction of luciferase was observed as the dose of 50 μM for **48** and **49** with 3.8 and 5.6 folds more than that of blank control [27]. The antioxidant capacity of **28** and **47**–**49** were evaluated by the modified 2,2′- azino-bis-(3-ethylbenzothiazoline-6-sulfonic acid) radical cation decolorization assay [27].

**Figure 4:**
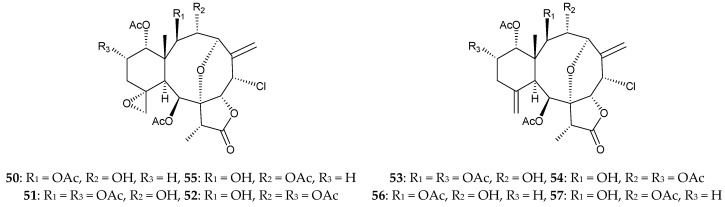
Structures of 3-deacetylpraelolide (**50**), 13-α-acetoxy-3-deacetylpraelolide (**51**), 13-α- acetoxy-2-deacetylpraelolide (**52**), 13-α-acetoxy-3-deacetyljunceellin (**53**), 13-α-acetoxy-2-deacetyl- junceellin (**54**), fragilide J (**55**), 3-deacetyljunceellin (**56**), and 2-deacetyljunceellin (**57**).

In 2017, Cheng et al. reported the occurrence of four pairs of chlorinated briarane derivatives, including five new metabolites, 3-deacetylpraelolide (**50**), 13-α-acetoxy-3-deacetylpraelolide (**51**), 13-α-acetoxy-2-deacetylpraelolide (**52**), 13-α-acetoxy-3-deacetyljunceellin (**53**), 13-α-acetoxy-2- deacetyljunceellin (**54**), along with three known metabolites, fragilide J (**55**) [28], 3-deacetyl- junceellin (**56**), and 2-deacetyljunceellin (**57**) [29], from *J. fragilis*, collected off the inner coral reef in Hainan Island, China (Figure 4), although briaranes **56** and **57** were obtained as a pair of inseparable mixture [30].

The structures of briaranes **50**–**54** were elucidated by spectroscopic methods in association with chemical conversion. The absolute configurations of briaranes **50** and **55** were further determined by acetylation of these two compounds to yield the same crystal product and analyses of X-ray crystal data of this compound by *Flack* parameter further confirmed the absolute configurations of briaranes **50** and **55** [30], although briaranes **56** and **57** existed in an inseparable mixture in CHCl_3_ at room temperature. Lowering the temperature to 4 °C resulted in the generation of a crystal, while the X-ray diffraction analysis using *Flack* parameter determined the crystal product to be in accordance with briarane **56**. Each pair of the isomers (**50**/**55**, **51**/**52**, **53**/**54**, and **56**/**57**) featured by dynamical interconversion through as acetyl migration in 1,2-diol, which was postulated to be generated under the formation of cyclic orthoacetate intermediated. In the mixture of briaranes **56** and **57**, increasing temperature gradients resulted in the variation of **56**/**57** ratio, while the ratio of **56**/**57** varied from 1:1 to 2:3 at 50 °C [30]. The mixtures of **50**/**55**, **51**/**52**, **53**/**54**, and **56**/**57** were tested for their inhibitory effects against NO production induced by LPS in RAW264.7 cells and these four components displayed inhibitory activities against NO production with the inhibition rates of 39.4%, 46.4%, 42.7%, and 36.3%, respectively, at a concentration of 50 μM [30].

**Figure 5:**
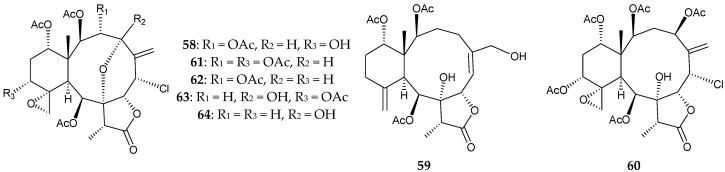
Structures of fragilides K (**58**) and L (**59**), gemmacolides V (**60**) and X (**61**), praelolide (**62**), juncins P (**63**) and ZI (**64**).

Two new briaranes, fragilides K (**58**) and L (**59**), along with five known chlorinated briaranes, gemmacolides V (**60**) and X (**61**) [31], praelolide (**62**) [7,14,16,32,33,34,35,36,37], and juncins P (**63**) [35] and ZI (**64**) [11], were obtained from a Formosan *J. fragilis* [38] (Figure 5).

Based on spectroscopic methods, the structures of briaranes **58** and **59** were elucidated and the cyclohexane rings in **58** and **59** were found to exist in chair and twist boat conformation, respectively. At a concentration of 10 μM, briaranes **59**, **61**, and **64** showed anti-inflammatory activity against the expression of pro-inflammatory protein inducible nitric oxide synthase (iNOS) to 49.13%, 36.22%, and 43.33%, respectively, and briaranes **60** and **61** elicited reduction of the pro-inflammatory protein cyclooxygenase-2 (COX-2) to 47.49% and 43.64%, respectively [38].

### 2.2. Junceella Gemmacea

**Figure 6:**
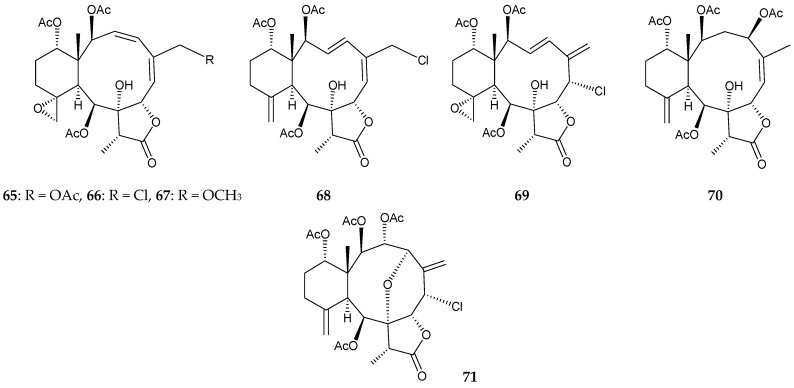
Structures of junceellolides M–P (**65**–**68**), junceellolides C (**69**) and D (**70**), and junceellin A (**71**).

Four new briaranes, junceellolides M–P (**65**–**68**), along with seven known briaranes, junceellolides A–D (**21**,**22**,**69**,**70**) [14] (the structures of briaranes **21** and **22**, please see Figure 2), junceellin A (= junceellin) (**71**) [7,14,16,34,35,36,37,39,40], praelolide (**62**) [7,14,16,32,33,34,35,36,37], and juncin ZI (**64**) [11] (the structures of briaranes **62** and **64**, please see Figure 5) were isolated from *J. gemmacea*, collected from the South China Sea [41] (Figure 6). The structures, including the absolute configurations, of new briaranes **65**–**68** were deduced on the basis of spectroscopic analyses, particularly with ECD experiments.

### 2.3. Junceella Juncea

**Figure 7:**
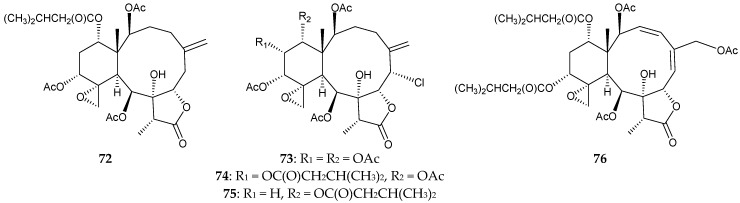
Structures of (1S*,2S*,8S*,9S*,10S*,11R*,12R*,14S*,17R*)-11,20-epoxy-14-(3-methyl- butanoyl)-2,9,12-triacetoxy-8-hydroxybriar-5(16)-en-18,7-olide (**72**), gemmacolides A (**73**) and B (**74**), and juncins H (**75**) and K (**76**).

Five 8-hydroxybriaranes, including a new briarane, (1S*,2S*,8S*,9S*,10S*,11R*,12R*,14S*,17R*)- 11,20-epoxy-14-(3-methylbutanoyl)-2,9,12-triacetoxy-8-hydroxybriar-5(16)-en-18,7-olide (**72**) along with four known metabolites, gemmacolides A (**73**) and B (**74**) [42,43], and juncins H (**75**) [44] and K (**76**) [45], were isolated from *J. juncea* collected from Tuticorin coast of the Indian Ocean (Figure 7) [46]. The structure of briarane **72** was established by spectroscopic data and **72** was found to exhibit activities against the fungi *Aspergillus niger*, *Candida albicans*, and *Penicillium notatum*. Briaranes **73** and **74** displayed activities against the bacteria *Bascillus pumilis* and *Escherichia coli*. While the briaranes **75** and **76** showed activities against *B. subtilis*, *B. pumilis*, *Proteus vulgaris*, and *E. coli* [46]. 

**Figure 8:**
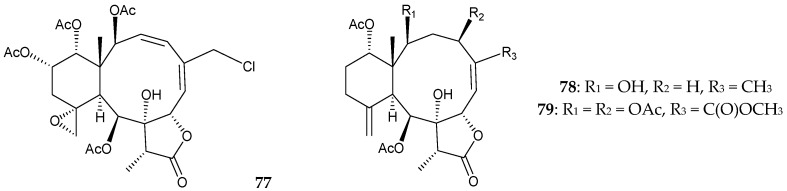
Structures of juncenolides M–O (**77**–**79**).

Furthermore, Chang et al. isolated three new briaranes, juncenlides M–O (**77**–**79**), from *J. juncea*, collected in the waters of Taiwan [47] (Figure 8). Structures of new briaranes **77**–**79** were established by spectroscopic methods. Briaranes **78** and **79** showed inhibitory activities against the release of elastase and **79** also exhibited inhibitory activity against the generation of superoxide anon [47].

### 2.4. Junceella sp. 

**Figure 9:**
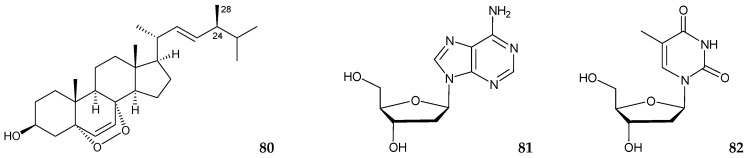
Structures of 5α,8α-epidioxy-24(ξ)-methylcholesta-6,22-dien-3β-ol (**80**), deoxyadenosine (**81**), and deoxythymidine (**82**).

Three known briaranes, junceellolide A (**21**) [14], praelolide (**62**) [7,14,16,32,33,34,35,36,37], and junceellin A (**71**) [7,14,16,34,35,36,37,39,40], (the structures of briaranes **21**, **62**, and **71**, please see Figure 2, Figure 5, and Figure 6, respectively), and a known sterol, 5α,8α-epidioxy-24(ξ)-methylcholesta-6,22-dien-3β-ol (**80**) [48] (Figure 9), were obtained from the ethanol extract of a gorgonian coral identified as *Junceella* sp., collected off the Vietnam Thu Island in May 2010 [49]. However, by assuming that enantiomeric series for sterols, the configuration at C-24 in **80** should be assigned as S*-form on the basis of the ^13^C NMR chemical shift of C-24 and C-28 [50]. Furthermore, two nucleosides, deoxyadenosine (**81**) and deoxythymidine (**82**) [51,52], were obtained from aqueous solution of this specimen. Structures of all isolates were established using spectroscopic data (Figure 9) [49].

In the cytotoxic activity test, briaranes **71** and sterol **80** exhibited weak cytotoxicity toward the THP-1 (human acute monocytic leukemia) tumor cells with IC_50_ values 55.4 and 130 μM, respectively. Sterol **80** also possessed weak clonogenic activity with INCC_50_ 53.3 μM toward THP-1. Moreover, sterol **80** produced an inhibition zone 12 mm in diameter against *Bacillus subtilis*. Briarane **62** inhibited weakly *Candida albicans*. Briaranes **21** and **71** and sterol **80** inhibited weakly *Vibrio parahaemolyticus* [49].

## 3. Conclusions

The natural products obtained from gorgonian corals belonging to the genus *Junceella* complied in this review indicated that the terpenoid derivatives, particularly briarane-type diterpenoids, are the major components of the natural products isolated. Of the 82 metabolites, 75 compounds (91.5%) are briarane-type diterpenoids. Of the briaranes, 50 compounds are halogenated (50/75 = 66.7%). Briarane-type natural products are a large family of natural products that are only isolated from marine organisms and the compounds of this type were suggested originally synthesized from the 3,8-cyclization of cembranoids by the host corals and not by their zooxanthellae [37,53,54]. Briarane-type diterpenoids continue to attract attention owing to their complex structures and potential biomedical activities.

Studies on the novel substances for biomedical use from the marine invertebrates originally distributed in the Indo-Pacific Ocean will play an important role in natural product research [55]. Marine natural products currently under clinical trials are limited. Based on the potential medical use and complex structures, it is very difficult to obtain enough material for further studies by chemical methods. How to make the best use of aquaculture technology to enhance in captivity mass production of raw materials needed for extraction of biomedical use marine natural compounds is very important in the future [56].
